# ELECTRONIC HEALTH RECORD SCREENING FOR PERSONS LIVING WITH DEMENTIA IN D-CARE

**DOI:** 10.1093/geroni/igac059.1208

**Published:** 2022-12-20

**Authors:** Mia Yang, Kristin Lenoir, Darcy McCurry, Katherine Currie, Nicholas Pajewski, Jeff Williamson

**Affiliations:** Wake Forest School of Medicine, Winston-Salem, North Carolina, United States; Center for Healthcare Innovation, Wake Forest School of Medicine, Winston Salem, North Carolina, United States; Department of Internal Medicine-Section on Gerontology & Geriatric Medicine, Wake Forest School of Medicine, Winston Salem, North Carolina, United States; Department of Internal Medicine-Section on Gerontology & Geriatric Medicine, Wake Forest School of Medicine, Winston Salem, North Carolina, United States; Department of Internal Medicine-Section on Gerontology & Geriatric Medicine, Wake Forest School of Medicine, Winston Salem, North Carolina, United States; Wake Forest School of Medicine, Winston-Salem, North Carolina, United States

## Abstract

Pragmatically identifying persons living with dementia (PLWD) for research is a persistent challenge, reflecting limitations in data elements. As part of the D-CARE study, the recruitment coordinator and analyst collaborated to create a screening algorithm to identify potentially eligible patients. A combination SAS/R script was applied on a monthly basis (Oct 2020-Dec 2021) to a clinical data warehouse, identifying living patients 50 years or older with ICD-10 codes for dementia or related conditions within the past two years, and an encounter within the past year. This process identified 6,478 unique patients, leading to the recruitment of 837 PLWD into the study (22% Black or Hispanic). Algorithmic components that enhanced recruitment efforts included the incorporation of data elements necessary to contact and enroll patients, formatting data consistent with reporting requirements, and tracking information on primary care provider, diagnosis dates with the set of providers that agreed to recruit for the trial.

